# Supplementary material for: Content-rich biological network constructed by mining PubMed abstracts
Source: BMC Bioinformatics. 2004 Oct 8;5:147. doi: 10.1186/1471-2105-5-147 (PMC528731; doi:10.1186/1471-2105-5-147)
Supplement: Additional File 2 — The original results of the above study (non-essential files are deleted to keep the file size under the limit set by BMC bioinformatics). [file 1471-2105-5-147-S2.bz2 › chilibotAdditionalFile2/dip20/84ID8139691E300/html/EPHA2_EFNA1.html]

 


 **EPHA2** and **EFNA1** 
  
Found 25 abstracts in PubMed, retrieved 20.  
 

 What does Google say? 
 PDF only 
| .edu only 

---

**Interactive relationship** (e.g. stimulation, inhibition, etc)

**Neutral relationship**- These results suggest that the Eck  [ **EPHA2** ]  B61  [ **EFNA1** ]  interaction, a target of regulatory peptides, plays a role in intestinal epithelial cell development, migration, and barrier function, contributing to homeostasis and preservation of continuity of the epithelial barrier.  Ref: 9357823 Am J Physiol, 1997
- B61  [ **EFNA1** ]  was also identified by Western blotting and ECK  [ **EPHA2** ]  binding activity with the use of a BIAcore binding assay in melanoma cell conditioned media.  Ref: 7780963 Cancer Res, 1995
- ECK  [ **EPHA2** ]  is a member of EPH receptor protein tyrosine kinase subfamily and human B61  [ **EFNA1** ]  has been identified as the ligand for ECK  [ **EPHA2** ]  recently.  Ref: 7675446 Oncogene, 1995
- In addition, a soluble form of LERK  [ **EFNA1** ]  7 will induce phosphorylation of eck  [ **EPHA2** ]  expressed in a human duodenum adenocarcinoma cell line.  Ref: 9245480 Cytokine, 1997
- It was recently discovered that soluble recombinant B61  [ **EFNA1** ]  could serve as a ligand for the Eck  [ **EPHA2** ]  receptor protein tyrosine kinase, a member of the Eph Eck  [ **EPHA2** ]  subfamily of receptor protein tyrosine kinases Bartley, T.D.  Ref: 7890684 J Biol Chem, 1995
- Ephrin A1  [ **EFNA1** ]  ligand was expressed in both tumor and endothelial cells, and  **EphA2**  receptor was localized primarily in tumor associated vascular endothelial cells.  Ref: 12370823 Oncogene, 2002
- Ephrin A1  [ **EFNA1** ]   **EFNA1**  is a GPI anchored ligand that preferentially binds to the receptor tyrosine kinase,  **EphA2** .  Ref: 11741747 Cancer Lett, 2002
- B61  [ **EFNA1** ] , a cytokine inducible endothelial gene product, is the ligand for the Eck  [ **EPHA2** ]  receptor protein tyrosine kinase RPTK.  Ref: 7536959 Science, 1995
- B61  [ **EFNA1** ]  has been identified recently by receptor affinity chromatography as a ligand for the Eph related receptor Eck  [ **EPHA2** ]  Bartley et al.  Ref: 8984203 J Neurosci Res, 1996
- These expression profiles of B61  [ **EFNA1** ]  in epithelial cells of various organs, developing skeletal system and dorsal root ganglia match those of ECK  [ **EPHA2** ] .  Ref: 7675446 Oncogene, 1995
- We now show that B61  [ **EFNA1** ]  can also exist as a cell surface glycosylphosphatidyl inositol linked protein that is capable of activating the Eck  [ **EPHA2** ]  receptor protein tyrosine kinase, the first such report of a receptor protein tyrosine kinase ligand that is glycosylphosphatidylinositol linked.  Ref: 7890684 J Biol Chem, 1995
- One member, LERK  [ **EFNA1** ]  7, is attached to the cell membrane via glycosyl phosphatidylinositol linkage and has been found to be a ligand for the eph family receptors hek, elk, eck  [ **EPHA2** ] , and rek.  Ref: 8661153 Genomics, 1996
- The Eck  [ **EPHA2** ]  RPTK was activated by tumor necrosis factor alpha TNF alpha through induction of B61  [ **EFNA1** ] , and an antibody to B61  [ **EFNA1** ]  attenuated angiogenesis induced by TNF alpha but not by basic fibroblast growth factor.  Ref: 7536959 Science, 1995
- B61  [ **EFNA1** ]  or LERK 1  [ **EFNA1** ] , recently identified as an ECK  [ **EPHA2** ]  ligand, stimulated the growth of ECK  [ **EPHA2** ]  expressing melanoma cell lines, its first identified biological activity.  Ref: 7780963 Cancer Res, 1995

**Non-interactive relationship** (e.g. studied together, co-existance, homology, etc.)

- This finding suggests the existence of an autocrine or paracrine loop involving activation of the Eck  [ **EPHA2** ]  RPTK by its inducible ligand B61  [ **EFNA1** ]  after an inflammatory stimulus, the net effect of which would be to promote angiogenesis, a hallmark of chronic inflammation.  Ref: 7536959 Science, 1995
- Expression and regulation of Eck  [ **EPHA2** ]  and its cognate ligand B61  [ **EFNA1** ]  were analyzed in the human colonic adenocarcinoma cell line Caco 2.  Ref: 9357823 Am J Physiol, 1997
- The expression of other members of this gene family, including ephrin B2,  **EphA2** , and ephrin A1  [ **EFNA1** ] , in a compartmentalized pattern within the Purkinje cell layer suggests a possible redundancy and or a compensation of EphA4 function in the segmental patterning of cerebellar Purkinje cells.  Ref: 11978390 Brain Res Dev Brain Res, 2002
- These results indicated that autocrine stimulation of  **EphA2**  by  **EFNA1**  could trigger an indirect growth signal by overcoming contact inhibition.  Ref: 11741747 Cancer Lett, 2002
- In order to better understand the roles of B61  [ **EFNA1** ]  ECK  [ **EPHA2** ]  signalling pathway in mammalian development, we have cloned rat and mouse B61  [ **EFNA1** ]  cDNA and examined the expression pattern during rat development.  Ref: 7675446 Oncogene, 1995
- These data suggest that ephrin A1  [ **EFNA1** ]  and  **EphA2**  play a role in human cancers, at least in part by influencing tumor neovascularization.  Ref: 11146556 Oncogene, 2000
- Functionally, stimulation of Eck  [ **EPHA2** ]  by B61  [ **EFNA1** ]  resulted in increased proliferation, enhanced barrier function, and enhanced restitution of injured epithelial monolayers.  Ref: 9357823 Am J Physiol, 1997
- Following addition of  **EFNA1**  Fc to HT29 cells, tyrosine hyperphosphorylation of  **EphA2** , E cadherin, and beta catenin were observed.  Ref: 11741747 Cancer Lett, 2002
- Strong tumor cell expression of FLT 4, ephrin A1  [ **EFNA1** ] , and  **EphA2**  was associated with increased melanoma thickness, and ephrin A1  [ **EFNA1** ]  staining was related to decreased survival P = 0.039.  Ref: 11891198 Am J Pathol, 2002
- Whether activation of  **EphA2**  by  **EFNA1**  is involved in aberrant growth or differentiation of cancer cells is currently not known.  Ref: 11741747 Cancer Lett, 2002
- Importance of vascular phenotype by basic fibroblast growth factor, and influence of the angiogenic factors basic fibroblast growth factor fibroblast growth factor receptor 1 and ephrin A1  [ **EFNA1** ]   **EphA2**  on melanoma progression.  Ref: 11891198 Am J Pathol, 2002
- Ephrin A1  [ **EFNA1** ]  and  **EphA2**  were also detected in both the vasculature and tumor cells of surgically removed human cancers.  Ref: 11146556 Oncogene, 2000
- Immunocytochemical staining demonstrated coexpression of Eck  [ **EPHA2** ]  and B61  [ **EFNA1** ]  in the same cells, suggestive of an autocrine loop.  Ref: 9357823 Am J Physiol, 1997
- Interestingly, the ligand for  **EphA2** , ephrin A1  [ **EFNA1** ] , is also regulated by p53.  Ref: 11641774 Oncogene, 2001
- ... the ephrin A1  [ **EFNA1** ]   **EphA2**  pathway might also be important for tumor cell proliferation and patient survival.  Ref: 11891198 Am J Pathol, 2002
- B61  [ **EFNA1** ] , a ligand for the Eck  [ **EPHA2** ]  receptor protein tyrosine kinase, exhibits neurotrophic activity in cultures of rat spinal cord neurons.  Ref: 8984203 J Neurosci Res, 1996
- Double labeling with anti CD34 antibodies demonstrated that both ephrin A1  [ **EFNA1** ]  and  **EphA2**  were expressed in xenograft endothelial cells and also tumor cells.  Ref: 11146556 Oncogene, 2000
- In addition, the expression patterns of B61  [ **EFNA1** ]  and Eck  [ **EPHA2** ]  during mouse ontogeny were determined by in situ hybridization.  Ref: 7890684 J Biol Chem, 1995
- To explore whether renal derived endothelial cells may distinguish LERK  [ **EFNA1** ]  2 from the angiogenic Eck  [ **EPHA2** ]  ligand, LERK 1  [ **EFNA1** ]  B61  [ **EFNA1** ] , and whether endothelial cells from different sources may distinguish among Eph receptor ligands, we compared HRMEC and human umbilical vein endothelial cell HUVEC responses in an in vitro capillary like assembly assay.  Ref: 8941926 Kidney Int Suppl, 1996
- EPH A2, or ECK  [ **EPHA2** ]  a receptor for ephrin A1  [ **EFNA1** ] , is ectopically expressed in most melanoma cell lines.  Ref: 10502726 Int J Cancer, 1999
- These neurotrophic effects of B61  [ **EFNA1** ]  were not observed in the presence of anti Eck  [ **EPHA2** ]  antibodies, indicating that these effects are likely to be mediated by the Eck  [ **EPHA2** ]  receptor.  Ref: 8984203 J Neurosci Res, 1996
- Role of B61  [ **EFNA1** ] , the ligand for the Eck  [ **EPHA2** ]  receptor tyrosine kinase, in TNF alpha induced angiogenesis.  Ref: 7536959 Science, 1995
- Cytokines interleukin 1beta IL 1beta, basic fibroblast growth factor, IL 2, epidermal growth factor, and transforming growth factor beta modulated steady state levels of Eck  [ **EPHA2** ]  and B61  [ **EFNA1** ]  mRNA and regulated Eck  [ **EPHA2** ]  activation as assessed by tyrosine phosphorylation.  Ref: 9357823 Am J Physiol, 1997
- **EphA2**  and ephrin A1  [ **EFNA1** ]  are members of the Eph family of receptor tyrosine kinases and ligands, which are implicated in a number of developmental processes.  Ref: 11641774 Oncogene, 2001
- Epithelial cell kinase  [ **EPHA2** ]  B61  [ **EFNA1** ]  an autocrine loop modulating intestinal epithelial migration and barrier function.  Ref: 9357823 Am J Physiol, 1997
- We have isolated both a ligand of the eph ligand family ELF, that we have named XELF a  [ **EFNA1** ] , and an eph related receptor, XE10, the likely homolog of the murine eck  [ **EPHA2** ]  Sek 2  [ **EPHA2** ]  receptor.  Ref: 8843391 Mech Dev, 1996
- By immunohistochemistry, the ephrin A1  [ **EFNA1** ]  ligand and one of its receptors,  **EphA2** , were detected throughout tumor vasculature.  Ref: 11146556 Oncogene, 2000
- Characterization of B61  [ **EFNA1** ] , the ligand for the Eck  [ **EPHA2** ]  receptor protein tyrosine kinase.  Ref: 7890684 J Biol Chem, 1995
- Protein B61  [ **EFNA1** ]  as a new growth factor expression of B61  [ **EFNA1** ]  and up regulation of its receptor epithelial cell kinase  [ **EPHA2** ]  during melanoma progression.  Ref: 7780963 Cancer Res, 1995
- Within one minute after stimulation of  **EphA2**  with its ligand, ephrin A1  [ **EFNA1** ] , the protein tyrosine phosphatase SHP2 is recruited to  **EphA2** .  Ref: 10655584 Nat Cell Biol, 2000
- The ephrin A1  [ **EFNA1** ]  ligand and its receptor,  **EphA2** , are expressed during tumor neovascularization.  Ref: 11146556 Oncogene, 2000
- Furthermore,  **EphA2**  was tyrosine phosphorylated in the xenograft tumors, indicating that it was activated, presumably by interacting with ephrin A1  [ **EFNA1** ] .  Ref: 11146556 Oncogene, 2000
- To examine the biological function of Eck  [ **EPHA2** ] , monolayer cultures of dissociated cells from embryonic rat spinal cord were treated with soluble B61  [ **EFNA1** ] .  Ref: 8984203 J Neurosci Res, 1996
- Remarkably, the oncogenic properties of  **EphA2**  contravene traditional dogma with regard to the oncogenic properties of a growth factor and its receptor tyrosine kinase while stimulation of  **EphA2**  by its ligand ephrin A1  [ **EFNA1** ]  results in  **EphA2**  autophosphorylation, the stimulation reverses the oncogenic transformation.  Ref: 12242697 Microsc Res Tech, 2002
